# Bacterial colonization at caesarean section defects in women of secondary infertility: an observational study

**DOI:** 10.1186/s12884-022-04471-y

**Published:** 2022-02-18

**Authors:** Isabel Hsu, Leonard Hsu, Sonam Dorjee, Chao-Chin Hsu

**Affiliations:** 1 Department of Obstetrics & Gynecology, National Taiwan University Hospital, Taipei, Taiwan; 2 Douglass Hanly Moir Pathology, Sydney, NSW Australia; 3 Taiwan United Birth-Promoting Experts Fertility Clinic, Tainan, Taiwan; 4 Department of Obstetrics & Gynecology, National Cheng Kung University Hospital, Tainan, Taiwan

**Keywords:** Caesarean section scar defect (CSD), Uterine niche, Bacterial colonization, Secondary infertility

## Abstract

**Background:**

Delayed childbearing has been noted in a high percentage of women with a previous Caesarean section (CS). Many women with CS scar defects (CSDs) present with clinical symptoms of irregular vaginal bleeding. The present study aimed to investigate bacterial colonies at CSDs in women suffering from secondary infertility.

**Methods:**

This observational study included 363 women with secondary infertility who visited the Assisted Reproduction Unit between 2008 and 2013. Among them, 172 women with a previous CS and 191 women with no previous CS were approached. The women with a previous CS had their CS operations in the past 1 to 14 years, with a mean of 3.5 years. The presence of CSDs was detected by vaginal ultrasonography. Bacteriology cultures of specimens taken from the uterine niches in those with CSDs were collected during Day 7 to Day 10 of the follicular phase. Specimens were obtained from the endocervical canal for bacterial culture in those without CSDs. The main outcome measure was the detection of the growth of bacterial colonies.

**Results:**

CSDs were found in 60.4% (96 of 159) of women with a previous CS. In women with a previous CS, bacterial colonies were identified in 89.6% (86 of 96) and 69.8% (44 of 63) of women with and without CSDs, respectively. In women with no previous CS, 49.7% (88 out of 177) of bacterial cultures of endocervical samples showed bacterial colony growth. Gram-positive cocci (*P* = 0.0017, odds ratio (OR) = 1.576, 95% confidence intervals (CI) -22.5 to − 5.4) and Gram-negative rods (*P* = 0.0016, OR = 1.74, CI − 20.8 to − 5.0) were the most commonly isolated bacteria and contributed to approximately 90% of all microorganisms found in those with a previous CS. In women with a previous CS, more Gram-negative rods were isolated (*P* = 0.01, OR = 1.765, CI − 27.2 to − 3.8), especially *Pseudomonas* species (*P* = 0.02, OR = 1.97, CI − 16.7 to − 1.0), in those with visible CSDs than in those without CSDs.

**Conclusions:**

Bacterial colonization at CSDs was found in a high percentage of women with secondary infertility.

## Background

Infertility is one of the major problems for women of childbearing age. Existing epidemiological studies have reported an association between Caesarean section (CS) and a lower percentage of subsequent pregnancies [1, 2]. Studies have shown that 42–50% of women who delivered via CS had no further children within 5 years, compared with 29% of those who delivered via spontaneous vaginal delivery [3, 4]. A relationship between CS and subfertility has been noted, where subfertility may both precede and be a consequence of CS [5]. Further complex associations, including social, psychological and pathophysiological factors, have also been discussed as causal mechanisms between CS and subsequent infertility [6]. A recent meta-analysis including 750,407 women showed an increased waiting time to the next pregnancy and risk of subfertility among women with a previous Caesarean delivery compared to women who delivered vaginally only [7]. Another meta-analysis including 85,728 women reported that CS, on average, reduced the probability of subsequent pregnancy by 10% in comparison to vaginal delivery [8]. However, the same authors further carried out a retrospective national population-based cohort study including over 1 million primiparous low-risk women and concluded that there is no, or only a slight, effect of CS on future fertility [9]. Their results suggested that unmeasured clinical and social factors during pregnancy and the intrapartum period that led to the decision to undergo a CS might explain the apparent effect of CS on future fertility.

The appearance of unhealed CS scar lesions, isthmoceles or uterine niches has brought extensive attention in the last two decades [10, 11]. The term CS scar defects (CSDs) was used to describe all anomalies characterized by a defect within the myometrium that reflects a breach at the site of a previous CS due to defective healing [12]. A relation between postmenstrual spotting and uterine niches has been reported in 2 prospective cohort studies [12]. Recent quality of life studies indicated that abnormal uterine bleeding, subfertility and abdominal pain were prioritized in women suffering from CSD niches [13, 14], and sexual and self-esteem were noted in focus group discussion studies [14]. To date, only a few studies have mentioned the relationship between the presence of uterine niches and secondary infertility [15, 16]. A very recent study showed that the presence of a CSD in women receiving in vitro fertilization (IVF) treatments, especially young women (age ≤ 35 years), significantly impaired the chances of subsequent pregnancy [17]. A larger study from an investigation of 1317 IVF cycles found that live birth rates were significantly lower among women with a previous CS than among women with a previous vaginal delivery (15.9 versus 23.3%, respectively [OR 0.63 95% CI 0.45–0.87]) and even lower live birth rates among women with CSDs (10.7%) [15]. It has been proposed that menstrual blood accumulation in uterine niches may interfere with the swimming of spermatozoa or impair embryo implantation, in turn impairing subsequent fertility [11]. However, the pathological mechanism for the formation of uterine niches and the outcome of infertility following CS have not been clearly defined.

In the present study, we studied the presence of bacterial colonies in uterine niches in women with secondary infertility. The results derived from the study might help to explain the causative factors for the formation of uterine niches and the sequelae affecting women with a previous CS.

## Methods

### Participants

**Fig. 1:**
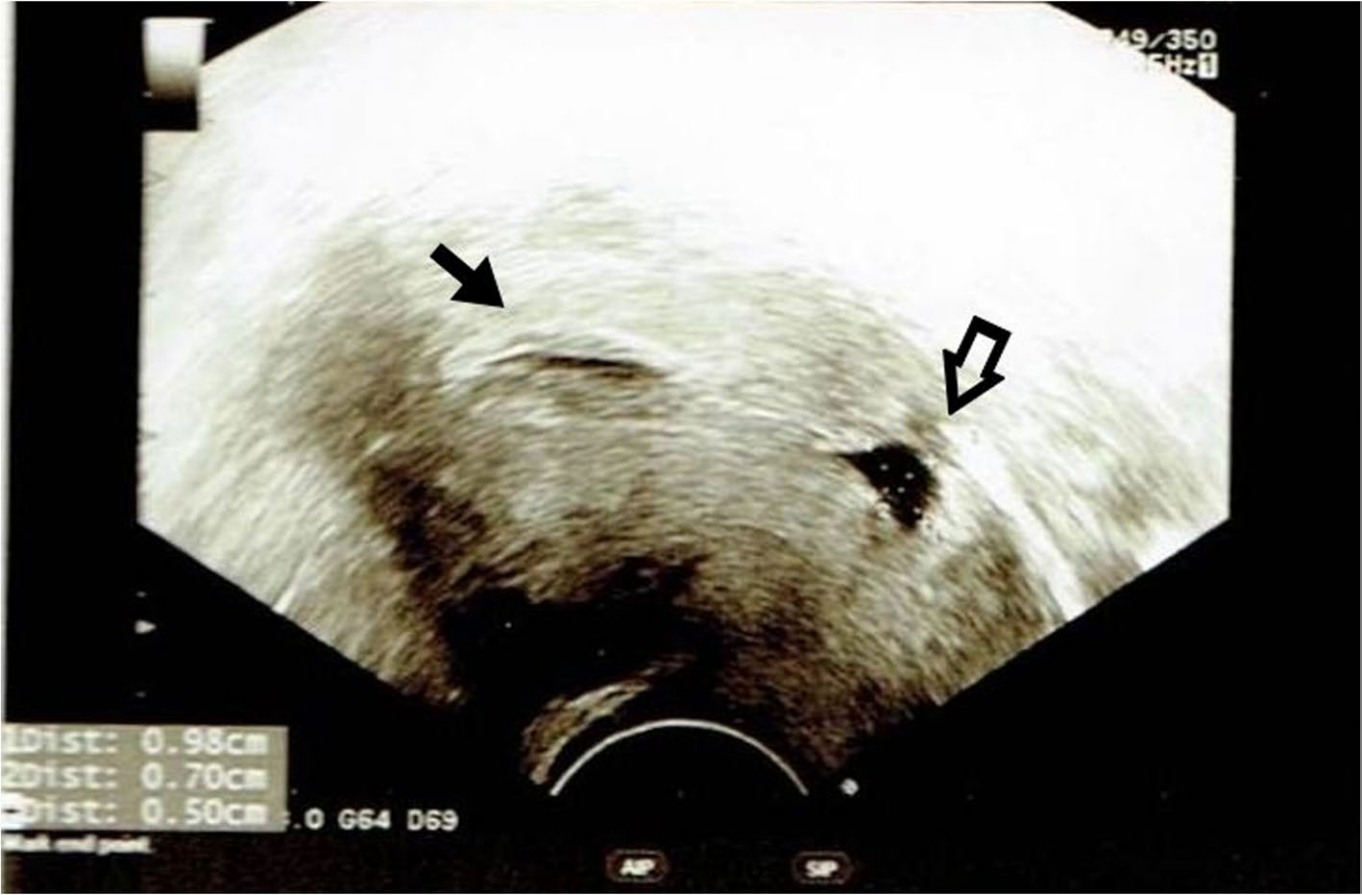
An ultrasonographic picture showing a sagittal view of the uterus and cervix. The left arrow indicates fluid accumulation in the uterine cavity, and the right arrow indicates a lesion measuring 9.8 × 7.0 mm at the lower uterine segment - a Caesarean section scar defect. A remaining myometrium thickness of 5 mm was measured

From October 2008 to December 2013, women with secondary infertility who visited the Taiwan United Birth-promoting Experts Unit for assisted reproduction treatments were approached to join this study. The exclusion criteria included women with pelvic inflammatory diseases, hydrosalpinx, uterine polyps, endometritis, cervicitis, vaginitis, antibiotic use 2 weeks prior to the study or intrauterine contraceptive devices in place during the 6 months prior to the study. Transvaginal ultrasonographic (TVS) scanning (5.0 MHz, Aloka SSD 1700, Aloka, Tokyo, Japan) was performed to locate the CS lesion around the lower uterine segments. The presence of a uterine niche (Fig. 1) was defined as an indentation at the site of a Caesarean scar representing a myometrial discontinuity that communicates with the uterine or cervical cavity over 2.5 mm [16]. Participants were divided into three groups: Group A,women who had a previous CS and a detectable uterine niche; Group B,women who had a previous CS but no detectable uterine niche; and Group C,women who presented with secondary infertility without a history of CS or any uterine lesions (the control group). The local ethical committees approved this study, and all subjects signed informed consent prior to enrollment. Each participant’s identification was based on a validated code in accordance with the “Reporting of studies Conducted using Observational Routinely collected health Data statement”.

### Intervention

Bacteriology cultures of specimens taken from the uterine niches in Group A were collected from Day 7 to Day 10 of the follicular phase. First, a thorough pelvic examination was performed to rule out active inflammatory diseases of the pelvic organs and lower genital tract. Second, a bivalve vaginal speculum was inserted into the vagina, and the secretions and contour of the vagina and cervix were inspected to rule out the presence of inflammation, ulcerations, and warty growth. Third, the vagina was cleansed thoroughly with isotonic sodium chloride solution, and any mucous content or bloody discharge in the cervix region was removed. Then, a cotton swab (BBL™ CultureSwab™ EZ and Plus, Becton Dickenson, le Pont de Claix, France) was used for sampling by inserting it through the cervical canal toward the uterine niche under abdominal sonographic guidance in Group A. The cotton swab was held at the lesion site for 5 seconds and was rotated against the site before withdrawal for bacterial isolation. Bacterial cultures of the endocervical canal approximately 2–3 cm proximal to the external os from subjects in Groups B and C were also obtained from Day 7 to Day 10 of the follicular phase. All ultrasonographic examinations and swabs taken were performed by the same investigator (C.C. Hsu). Collected samples were incubated at 35 °C overnight with appropriate media including BAP/EMB Biplate (BBL™ Trypticase™ Soy Agar with 5% Sheep Blood/Levine EMB Agar, BD), BD Chocolate agar plate (GC II Agar with IsoVitaleX and Blood Agar No. 2 Base), Chrom ID™ Candida Agar (CCA, bioMérieux, Marcy-l’Étoile, France), a CNA agar plate (BD Columbia CNA Agar with 5% Sheep Blood), and Thio broth CDC AnBAP PEA agar plate (BD BBL™ CDC Anaerobe 5% Sheep Blood Agar with Phenylethyl Alcohol). Once the culture was identified to be positive, Gram staining and subculturing were undertaken. BAP/EMB Biplates and Gram staining were used for bacterial strain identification. The disk diffusion antimicrobial susceptibility method was used for antibiotic susceptibility tests. Specific antibiotics were given to the positive bacterial strains identified. Repeated cotton swab samplings were taken for microbiology tests under consent in some participants who did not conceive after 3–6 months of follow-up. The primary outcome of the present study was the identification of the presence and the variability of bacterial colonies found in the CSDs of infertile women.

### Statistical analysis

The data were analyzed using the chi-square test for differences between two proportions. All continuous data are expressed as the mean value ± standard deviation (SD). Statistical calculations were performed using JMP software, and a *P* value < 0.05 was considered statistically significant. In view of the mode of data distribution, 95% confidence intervals (CI) were presented.

## Results

**Fig. 2:**
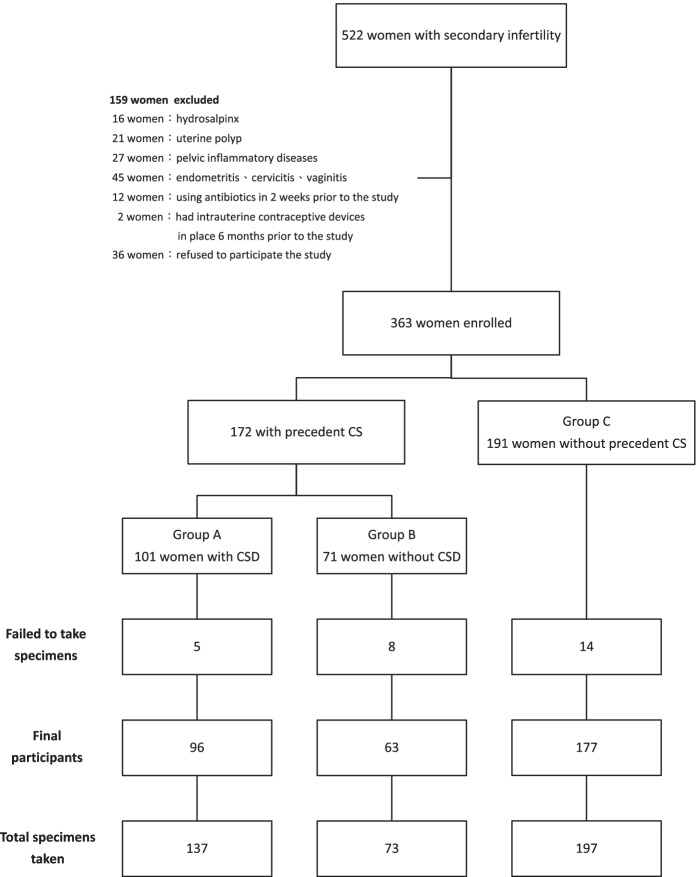
Flow chart identification of participants in this study. CSD: Caesarean section defect

Among 522 women approached, 159 women were excluded, as indicated in Fig. 2. Among 363 women enrolled, 27 were further excluded from this study due to difficulty obtaining an adequate amount of specimen owing to stenotic endocervical canals. In total, 137, 73, and 197 specimens were taken from uterine niches or endocervixes for bacterial culture from 96, 63, and 177 participants in Groups A, B, and C, respectively (Fig. 2). The demographic characteristics of the participants are described in Table 1. No differences were noted in patient age or the duration of infertility among the three study groups or in whether the previous CS was performed at a hospital-based system or a local obstetrics clinic (*P* = 0.0919, CI 12.70 to − 34.70). A uterine niche was seen in 60.4% (96 of 159) of women with a history of CS. Most women (~ 70%) with a uterine niche experienced persistent period flow and/or postmenstrual irregular vaginal darkish discharge. Many women also experienced lower abdominal pain and/or twitching sensations.

**Table 1:** Demographic characteristics of women with secondary infertility

		With uterine niches post-CS	Without uterine niches post-CS	Without CS
**Number of subjects**		96	63	177
**Age (years)**		24–45 (34.9 ± 4.7)^a^	24–47 (34.4 ± 4.6)	21–45 (32.5 ± 4.7)
**No. of CSs**		1–4 (1.44 ± 0.72)	1–2 (1.16 ± 0.22)	–
**Duration of infertility (years)**		1–13 (3.8 ± 3.2)	1–14 (3.3 ± 3.1)	1–18 (4.7 ± 3.5)
**Retroverted uterus**		56 (58.3%)	29 (46.0%)	75 (42.3%)
**CS performed at**	**Obstetrics clinic**	49 (62.8%)	29 (37.2%)	–
	**District hospital**	29 (63.0%)	17 (37.0%)	–
	**University hospital**	18 (51.4%)	17 (48.6%)	–
**Prolonged darkish vaginal flow**		67 (69.8%)	9 (14.3%)	–
**Bacteria colony identified (per case)**		86 (89.6%)	44 (69.8%)	88 (49.7%)
**Bacteria colony identified (per specimen)**		127/137 (92.7%)	54/73 (74.0%)	108/197(54.8%)
**Mean No. microorganisms identified**		1.27	1.3	1.28
**More than one microorganism identified**		26/137 (19.0%)	11/73 (15.1%)	16/197 (8.1%)

^a^ mean ± SD; *CS* Caesarean section

There was a significant difference in the proportion of women from whom a single bacteria was isolated from samples between those with a previous CS compared with those in the control group (*P* <  0.001, odds ratio (OR) = 1.35, CI 15.0 to 30.6) and between Group A and Group B, i.e., the presence of a CSD or not (*P* <  0.001, OR = 1.23, CI 7.2 to 27.6) (Tables 2 & 3). An average of 1.28 specific microorganisms, excluding normal mixed flora, was identified in each specimen taken from the subjects.

**Table 2:** Aerobic bacteria isolated from secondary infertility patients with a previous Caesarean section

Aerobic bacteria	CSD niche (+)		CSD niche (−)		***P*** value
Number *	151		79		
Number **	126		54		
**Gram (+) cocci**	57	37.75%	31	39.24%	NS
*Streptococcus* A	0	13.25%	1	8.86%	NS
*Streptococcus* B	14		5		
*Streptococcus* D	1		0		
*Streptococcus* G	1		0		
*Streptococcus* groups α, γ	4		1		
*Enterococcus*	23	15.23%	12	15.19%	NS
*Staphylococcus aureus*	4	9.27%	4	15.19%	NS
*Staphylococcus* coag (−)	10		8		
**Gram (+) rods**	3	1.99%	4	5.06%	NS
**Gram (−) cocci**	0	0%	0	0%	
**Gram (−) rods**	54	35.76%	16	20.25%	< 0.01
*Escherichia coli*	24	15.89%	8	10.13%	NS
*Klebsiella pneumonia*	3	1.99%	2	2.53%	NS
*Proteus mirabilis*	4	2.65%	0	0.00%	NS
Other *Enterobacter* spp.	0	0%	1	1.27%	NS
*Pseudomonas aeruginosa*	4	15.23%	0	6.32%	< 0.01
*Pseudomonas* spp.	8		1		
*Burholderia cepacia*	10	0.66%	3	0.00%	NS
*Stenotrophomonas maltophilia*	1		1		
***Gardnerella vaginalis***	1		0		
**Yeast**	9	5.96%	1	1.27%	NS
**Others**	2	1.32%	2	2.53%	NS
**Normal mixed flora**	15	9.93%	6	7.59%	NS
**No growth**	10	7.94%	19	24.05%	< 0.001

*CSD* Caesarean section scar defect

Number*: total numbers include specific pathogenic organisms isolated plus numbers of normal mixed flora and no growth

Number**: numbers of specific pathogenic organisms isolated. No repeated counting of the numbers in those with repeated isolation of the same bacteria in a single individual

%: the ratio of specific bacteria isolated out of the total number (Number*)

**Table 3:** Aerobic bacteria isolated from secondary infertility patients with and without a previous Caesarean section

Aerobic bacteria	With CS		Without CS		***P*** value
Number *	230		206		
Number **	180		104		
**Gram (+) cocci**	88	38.25%	50	24.27%	0.001
*Streptococcus* A	1	11.72%	0	9.71%	NS
*Streptococcus* B	19		16		
*Streptococcus* D	1		2		
*Streptococcus* G	1		1		
*Streptococcus* groups α, γ	5		1		
*Enterococcus*	35	15.22%	17	8.25%	0.02
*Staphylococcus aureus*	8	11.31%	1	6.80%	NS
*Staphylococcus* coag (−)	18		13		
**Gram (+) rods**	7	3.04%	1	0.49%	0.04
**Gram (−) cocci**	0	0.00%	0	0%	
**Gram (−) rods**	70	30.43%	36	17.48%	< 0.001
*Escherichia coli*	32	13.91%	22	10.68%	NS
*Klebsiella pneumonia*	5	2.17%	2	0.97%	NS
*Proteus mirabilis*	4	1.74%	2	0.97%	NS
Other *Enterobacter* spp.	1	0.43%	1	0.49%	NS
*Pseudomonas aeruginosa*	4	12.17%	0	4.36%	< 0.01
*Pseudomonas* spp.	9		1		
*Burholderia cepacia*	13		7		
*Stenotrophomonas maltophilia*	2		1		
***Gardnerella vaginalis***	1	0.43%	0	0.00%	NS
**Yeast**	10	4.35%	13	6.31%	NS
**Others**	4	1.74%	4	1.94%	NS
**Normal mixed flora**	21	9.13%	29	14.08%	NS
**No growth**	29	12.61%	73	35.44%	< 0.001

Number*: total numbers include specific pathogenic organisms isolated plus numbers of normal mixed flora and no growth

Number**: numbers of specific pathogenic organisms isolated. No repeated counting of the numbers in those with repeated isolation of the same bacteria in a single individual

%: the ratio of specific bacteria isolated out of the total number (Number*)

In those with a previous CS, compared with the control group, Gram-positive cocci (*P* = 0.0017, OR = 1.576, CI − 22.5 to − 5.4) and Gram-negative rods (*P* = 0.0016, OR = 1.74, CI − 20.8 to − 5.0) were the most commonly isolated bacteria and contributed to approximately 90% of all microorganisms (Table 3). Among Gram-positive cocci, Group B *Streptococcus* and *Enterococcus* species were more frequently (nearly 90%) isolated from all women examined and were more frequently isolated from women with a previous CS (*P* = 0.02, OR = 1.7, CI − 12.9 to − 0.99). Among Gram-negative rods, the *Pseudomonas* species were abundant (*P* = 0.01, OR 1.8, CI − 12.8 to − 2.7) in women with a previous CS. Comparing samples from Groups A and B for those with a previous CS, there was a significant difference in Gram-negative rods isolated (*P* = 0.01, OR = 1.765, CI − 27.2 to − 3.8), especially *Pseudomonas* species (*P* = 0.02, OR = 1.97, CI − 16.7 to − 1.0). Almost all of the same bacterial strains were cultured from those that underwent repeated sampling.

Yeast colonization, including colonization by *Candida albicans*, *Candida glabrata*, and other fungi, accounted for approximately 2 to 6% of women with a previous CS and up to 6% of women in the control group. Other bacteria isolated, included *Acinetobacter lwoffii, Citrobacter freundii, Citrobacter diversus*, and *Chryseobacterium* sp., which are not listed in Tables 2 and 3. A few anaerobes were isolated as well, including *Peptostreptococcus* spp.*, Clostridium* spp.*, Eubacterium* spp.*, Bacteroides* spp.*,* and *Fusobacterium* spp.

## Discussion

### Main findings

A great percentage (60%) of women with a previous CS suffered from secondary infertility partly due to complications arising from uterine niches. Bacterial colonies were identified in 89.6 and 69.8% of the participants with a previous CS with and without uterine niches, respectively. Excluding the nonspecific normal mixed flora, we isolated an average of 1.28 aerobic bacteria from the specimens taken. Thus, bacterial colonies were found at the CSD in most women with a previous CS who suffered from secondary infertility.

### Strengths and limitations

The participants recruited in this study had undergone a CS within a range of 1 to 14 years, with a mean duration of 3.5 years. To our knowledge, a large-scale investigation of bacterial strains in women with postpartum uterine lesions lasting longer than 1 year has never been reported before, except in a recent report of abscesses in the isthmocele of a woman 10 years after a CS [18]. The presence of bacteria in uterine niches may explain common CS sequelae, including lower abdominal pain, persistent vaginal discharge, and even late sequelae, such as increased morbidities in future pregnancy and subfecundity. A few limitations exist for the present study. First, we did not have information on whether the previous CS was planned or an emergency CS. Some potential factors affecting the lower uterine segment, such as the duration of labor and dilatation, were found to influence the development of a niche [19]; both having a CS and having subsequent infertility may be related to antenatal or intrapartum infection. Additionally, antenatal and intrapartum histories for all participants were not available in this study. Second, bias might exist both in the ultrasonography findings and when taking swabs, as the investigators were not blinded to whether a woman had a CS. Third, our unit is an assisted conception unit and did not have access to women with a previous CS who were not infertile. Ideally, a random sample of women with a history of CS should be recruited as study subjects to prevent selection bias. Another group of women who have experienced only vaginal birth may also be recruited to distinguish the effects of CS [19]. Fourth, due to the lack of standardized sampling techniques, which might lead to difficulty avoiding bacterial contamination from the lower genital tract, and the limited facilities at the laboratory to isolate microorganisms, we did not have sufficient data for the anaerobic bacteria isolated in this study. In addition, the sites for the specimens taken for bacterial culture were different, including the CS niche and endocervical canal, in those with and without a CSD, respectively. The bacterial strains cultured from the lower uterine segment where the CSD niche was located could differ from those from the endocervical canal. However, technical difficulty existed in taking samples from the lower uterine segment in women without a CSD niche. However, specimens from the most nearby tissue of the endocervical canal were taken to be compared to those taken from CSD niches. Bacterial strains at the cervix and endocervical canal of women with CSD niches were also not investigated. Another limitation of this study was that the participants were recruited from October 2008 to December 2013, a relatively long time ago, and almost one-third of initially recruited participants were excluded due to various factors. Although profuse bacterial strains were cultured from CSD niches, whether bacterial colonization was the effect or one of the major causative factors of CSD niche formation was not investigated in the present study. One study revealed a notable obstacle in investigating microorganisms in the female genital tract, including the uterine cavity, with a matching rate of 56.92% between the microbial culture and the real-time polymerase chain reaction results. However, the results among the 3 classic techniques, including histological examination, hysteroscopy and endometrial culture, in the diagnosis of chronic endometritis were only 20% concordant [20]. Microbial culture, the most reliable of the 3 classic methods, also presents some limitations, mainly due to the contamination of the microbial culture with skin contact or environmental bacteria and the failure to grow and isolate nonculturable bacteria. Despite commonly found in the female genital tract and may be a co-factor in bacterial vaginosis, the evidence that genital mycoplasmas cause lower genital tract disease in women remains sparse [21, 22]. Routine screening of asymptomatic men and women or routine testing of symptomatic individuals for *Mycoplasma hominis*, and *Ureaplasma urealyticum* is not recommended from - a position statement of the European sexually transmitted infections (STI) Guidelines Editorial Board [23]. In the present study, we thus did not perform the culture of *Mycoplasma hominis*, and *Ureaplasma urealyticum* in women receiving CS.

### Interpretation

#### Sterility in the uterine cavity?

The uterine cavity has long been regarded as microbiologically sterile under normal, healthy circumstances, except at the time of labor, immediately following delivery or miscarriage, and under the use of intrauterine contraceptive devices [24, 25]. In contrast, the cervix and vagina are normally colonized by a variety of microorganisms. The barrier preventing the upward spread of bacterial infection is mainly at the uterine cervix. In established pregnancies, the cervical barrier is created by the naturally compacted space of the amnion and chorion across the internal os. The biochemical barrier of the cervix is created by lysozyme, an enzyme capable of hydrolyzing the beta 1–4 peptidoglycan linkage of microorganisms, allowing osmotic lysis [26]. Ascending vaginal infection is thought to be the most common route by which bacteria gain access to the uterine cavity [27]. The effects of CS, especially the location of the uterine incision, may compromise the cervical barrier and lead to infection ascending to the upper genital tract by bacteria that do not normally inhabit this area [25]. A recent study on ascending vaginal infection in antepartum women using bioluminescent bacteria in real-time bioluminescence imaging showed that bacteria first spread within the choriodecidual space and then to the placenta and fetal membranes [28]. Histological examination of specimens removed from the CS scar revealed the presence of inflammatory infiltration in 70% of the cases [11, 29]. In the present study, a great percentage of women had bacterial colonization in uterine niches. Thus, the natural body defense mechanism at the cervix, including the physical barrier of the internal os and the biochemical cervical barrier, might have been disrupted in our study participants.

More recent studies have indicated the presence of microorganisms in the uterine cavity [30–32]. Colonization with potentially pathogenic organisms was found in one-quarter of the uterine cavity in a prospective study using hysterectomy samples [30]. A more recent analysis of endometrial microbiota in intrauterine adhesion by high-throughput sequencing showed that the uterine cavity is not sterile and contains various bacteria [31]. The ‘sterile womb’ hypothesis has also been challenged by recent studies using next-generation sequencing (NGS) analysis of the genomes of microorganisms, which overcomes two common limitations of traditional culture-based microbe characteristics, nonculturability and genomic diversity [32], opening a new research field in reproductive medicine [33]. The balance of microecology in the female reproductive tract plays a key role in health. Evidence suggests that changes in the composition and distribution of the endometrial microbiota are related to endometrial diseases such as endometrial polyps, endometrial cancer, and infertility [23, 34, 35]. Studies have shown that the numbers of *Gardnerella*, α-*Streptococcus*, *Enterococci* and *Escherichia coli (E. coli)* were significantly higher in endometrial samples from women with endometriosis than in those without endometriosis [36]. The high prevalence of virulent and resistant uropathogenic *E. coli* strains in the upper vagina of infertile women with a history of urinary tract infections is suggestive of the important role of these pathogens in female infertility [37]. Thus, microbial dysbiosis present in the uterine cavity may be one of the causative factors that leads to gynecological disorders, including infertility.

#### Bacteria strains isolated from the CS niches

In the present study, Group B *Streptococcus* and *Enterococcus* species were more frequently isolated (nearly 90%) among Gram-positive cocci, and *E. coli* and *Pseudomonas* species were profound among Gram-negative rods. Similar findings were noted from a study on the prevalence of chronic endometritis in infertile patients [38], in which *Streptococcus* species were the most abundant bacteria detected (47%), followed by *Enterococcus* species (15%), *E. coli* (12%), *K. pneumoniae* (5%), *Staphylococcus* species (3%), and *M. hominis*(2%). These findings are also consistent with previously reported microbial culture data [20, 34]. The findings, however, were different from those of other studies in which *Lactobacillus* species dominated in healthy women and *Acinetobacter*, *Pseudomonas* and *Comamonadaceae* dominated in women who had undergone gynecological operations [33].

Previous studies have shown that aerobic Gram-negative rods are causally involved in 10–20% of cases of endometritis following cesarean section. Among aerobic Gram-negative rods, *E. coli* is most commonly isolated in both genital and blood cultures. *Klebsiella pneumoniae* and *Proteus mirabilis* rank second and third, respectively, followed by *Enterobacter* species. *Pseudomonas* species account for fewer than 0.6% of genital isolates [35]. However, we found that both *E. coli* and *Pseudomonas* species predominate among the Gram-negative rod colonies. It has been reported that highly virulent organisms are less frequently found in swabs taken from the uterine cavity and CS wounds [39, 40]. Recent NGS studies have indicated the potential molecular functions of the endometrial microbiome linked to cell metabolism, motility, genetic information, the immune system, and signaling processes [41, 42]. Some strains of *Klebsiella* can produce virulence factors such as lipopolysaccharide (LPS), which acts on Toll-like receptor 4 (TLR4) to induce fibrosis and inflammation [43]. *Pseudomonas aeruginosa* can produce a biofilm in the uterus, and the host immune response is modulated focally around areas with biofilms [44]. Whether the microorganisms isolated in the present study affected or were one of the major causative factors of CSD niche formation requires further investigation.

#### Formation of the uterine niche

A CS defect was defined as an indentation with a depth of > = 2 mm at the site of CS evaluated by TVS by means of a modified Delphi consensus [45]. In a random population of women with a history of CS, the prevalence of a niche ranged from 24 to 70% and 56 to 84% when assessed by TVS scanning and sonohysterography, respectively [19]. Women with only one CS had a 35–61% chance of developing a CSD, while the risks were 76–81% and 88–100% after two and three CSs, respectively [39, 40]. In a prospective cohort study among women undergoing hysteroscopic sterilization, a uterine niche could be detected by hysteroscopy in 75% of women with a previous CS [46]. Thus, the presence of CSDs existed in the majority of women with a previous CS.

Four hypotheses have been raised for the formation of CSDs: (1) a cervical location of the uterine incision induces impaired wound healing; (2) incomplete closure of the uterine wall; (3) surgical activities that may induce adhesion formation and, as a consequence, induce impaired wound healing due to counteracting forces on the uterine scar; and (4) patient or disease-related factors, such as peripartum infections, elevated body mass index and diabetes, that impair wound healing [11, 22, 47]. Moreover, uterine niche development was significantly associated with repeated CSs, prelabor rupture of membranes, a short operation time, and the extent of cervical dilatation at the time of CS [48]. A systematic review that included 20 randomized controlled trials was performed to evaluate single- versus double-layer suturing in relation to adverse outcomes and the prevalence of uterine niches [49]. The majority of the studies showed that double-layer uterine closure using nonlocking sutures might result in a thicker residual myometrium and potentially a lower prevalence of niches. A lower segment incision with the double-layer suture technique is routinely employed for CS wound closure in Taiwan; thus, this issue might not be a causative factor in our patients presenting with uterine niches. The prevention of peripartum infection, the reduction of CS time, the reduction of blood loss and more careful uterine closure are needed to decrease the risk of CSD development [50]. Prophylactic antibiotics given to all women undergoing elective or non-elective Caesarean section is clearly beneficial for women in reducing two thirds to three quarters on the occurence of endometritis and wound infection from a Cochrane Database Systemic Review [51].

Metabolic end products such as enzymes and toxins produced by the localized growth of pathogenic or normal flora might accumulate in uterine niches, causing a disruption of tissue structure and damage [52]. However, we did not know whether bacterial colonization is an effect or one of the major causative factors of CSD niche formation. As mentioned above, most of the microorganisms isolated in this study were highly virulent. Thus, future studies are mandatory to clarify the correlation between chronic bacterial colonization and the formation of uterine niches.

#### Uterine niches and secondary infertility

Approximately 30–40% of women with a previous CS with uterine niches suffered from secondary infertility [15, 16]. Other studies have shown that the risk of infertility among these women is estimated to range from 4 to 19% [9, 47]. The persistence of menstrual blood in uterine niches may negatively influence mucus quality, obstruct sperm transport through the cervical canal, affect sperm quality or eventually interfere with embryo implantation [19, 29]. The cytotoxicity of excess iron after hemoglobin degradation in the uterine cavity [53] may be toxic to the embryo or impair its implantation owing to disturbed endometrial receptivity or the alteration of the uterine microbiota [20]. However, evidence in support of a pathophysiological mechanism for infertility following CS remains inconclusive [6]. Significant pathological changes, including a distortion and widening of the lower uterine segment, congested endometrium, polyps, lymphocytic infiltration, residual suture material, capillary dilatation, free red blood cells, fragmentation and breakdown of the scar endometrium, and iatrogenic adenomyosis, may all be contributing causative factors interfering with successful conception [10, 12]. A recent report showed that the endometrial cavity is not sterile and that the colonization of the uterine cavity with non-*Lactobacillus*-dominated bacteria affects the success of in vitro fertilization, pregnancy rates, and live births [54]. For bacteria-colonized uterine niches, the production of toxic products from localized microorganisms might be relevant to the fecundity of women with CSDs. Surgical treatment of uterine niches has been shown to be effective in improving reproductive outcomes [55]. Uterine niches may also increase the risk of issues during embryo transfer in IVF treatments [56]. Embryo transfers performed on patients with a CSD took an average of 30 s longer and were more likely to accumulate blood or mucus on the catheter [56]. The intermediate roles of the niche on fertility outcomes have recently been hypothesized and included: the accumulation of mucus and old blood in the niche may impair sperm penetration; the accumulation of intrauterine fluid may impair implantation; impaired accessibility for embryo transfer due to a distorted anatomy caused by the large niche in combination with a strongly retroflexed uterus; altered immunobiology and/or increased inflammation in uterine cavity; and the interference with sexual intercourse due to niche-related gynecological symptoms [15]. Although the accumulation of fluid and mucus in a CSD niche may facilitate bacterial growth, reducing the chances of a successful pregnancy [54], examining the microbial composition of niches or inflammatory processes in women with a CSD niche has not been reported [15]. Whether the chronic localized bacterial colonies observed in the present study might be a causative factor for the formation of uterine niches and secondary infertility requires further study.

#### Different results when the previous CS was performed in a hospital-based system

In Taiwan, it has long been a tradition that many expecting women obtain prenatal care and deliver their infants at local obstetrics clinics. Thus, almost half of our participants had their previous CS at a local obstetrics clinic. In the present study, there was a trend of a lower incidence of a uterine niche in women who had undergone their CS at university-based medical centers. There might be confounding factors of population characteristics in women who underwent CS at university-based medical centers, including chronic diseases, such as diabetes mellitus and cardiovascular diseases, or high-risk pregnancies, such as those associated with preeclampsia, placenta previa, and preterm labor. It has been noted that the incidence of CSDs was higher in women with high-risk pregnancies [11, 19]. However, in the present study, we found that the incidence of CSDs was lower in women who underwent their CS at university-based medical centers. In addition, the protective processes and septic procedures might be more stringent at university medical centers, which could be used to explain the result, as there was no difference in the use of prophylactic antibiotics at different hospitals or clinic-based CS performance in Taiwan. Large variations regarding the level of service and the probability of complications across US hospitals have been reported [57]. Regarding the size of medical units carrying out CSs, the findings from a Canadian study indicated that rural hospitals had lower odds of surgical errors and complications, whereas hospitals with high bed numbers had greater odds of errors and complications than medium bed number hospitals [58]. More recent studies have shown that the training status and experience of physicians performing CSs and the caseload volume of the medical institute play important roles in maternal postsurgical complications after CS [59]. Both individual- and hospital-level factors were associated with surgical errors and complications following CS. Approximately 27% of the odds of surgical error during CS could be explained by between-hospital differences [60]. Thus, future studies on post-CS complications to explore different indicators of physician experience, as well as the role of an individual’s socioeconomic level, are highly demanded for future patient safety initiatives.

## Conclusions

This study found the presence of various microorganisms in uterine niches in broad postpartum timeframes. The identification of potential risk factors and the clinical relevance of the presence of bacterial colonies in uterine niches, especially for fecundity, should be further investigated.

## Data Availability

The dataset supporting the conclusions of this article will be available by proper request to the corresponding author.

## Abbreviations

CS: Caesarean section
CSD: Caesarean section defects
TVS: Transvaginal ultrasonographic
SD: Standard deviation
CI: 95% confidence intervals
OR: Odds ratio
*E. coli*: *Escherichia coli*
